# Comparison between remifentanil and dexmedetomidine for sedation during modified awake fiberoptic intubation

**DOI:** 10.3892/etm.2015.2288

**Published:** 2015-02-13

**Authors:** HUI-HUI LIU, TAO ZHOU, JIAN-QI WEI, WU-HUA MA

**Affiliations:** 1 Department of Anesthesiology, The First Affiliated Hospital of Guangzhou University of Chinese Medicine, Guangzhou, Guangdong 510405, P.R. China; 2 Department of Otolaryngology, Union Hospital, Huazhong University of Science and Technology, Wuhan, Hubei 430022, P.R. China

**Keywords:** remifentanil, dexmedetomidine, local anesthesia, awake fiberoptic intubation

## Abstract

Cricothyroid membrane injections and the application of a coarse fiberoptic bronchoscope (FOB) below the vocal cords for topical anesthesia have a number of limitations for certain patients. Thus, the aim of the present observational study was to assess the effect of a novel modified topical anesthesia method using the effective sedation drugs, remifentanil (Rem) or dexmedetomidine (Dex), during awake fiberoptic orotracheal intubation (AFOI). In total, 90 adult patients, who had been classified as American Society of Anesthesiologists I–II, were included in the study. The patients had anticipated difficult airways and were to undergo orotracheal intubation for elective surgery. The patients were enrolled in the double-blinded randomized pilot study and received Rem or Dex for sedation during the modified AFOI procedure. The two groups received 2% lidocaine for topical anesthesia via an epidural catheter, which was threaded through the suction channel of the FOB. The main clinical outcomes were evaluated by graded scores representing the conditions for intubation and post-intubation. Additional parameters analyzed included airway obstruction, hemodynamic changes, time required for intubation, amnesia level and subjective satisfaction. All 90 patients were successfully intubated using the modified AFOI technique. The comfort scores and airway events during intubation did not significantly differ between the two groups. However, the Rem group experienced less coughing, and less time was required for tracheal intubation when compared with the Dex group. No statistically significant differences were observed in the changes to the mean arterial pressure and heart rate at any time point between the two groups. Therefore, the current study demonstrated that the modified AFOI method is feasible and effective for difficult airway management, and that Dex and Rem exhibit similar efficacy as adjuvant therapies.

## Introduction

Awake fiberoptic orotracheal intubation (AFOI) is used in patients with expected difficult airways. Adequate topical anesthesia and sedation techniques are important during the procedure in order to maintain the patient’s airway and minimize discomfort. Optimal conditions for modified AFOI should enable the patients to be cooperative, comfortable and have blunted airway reflexes, particularly when difficult laryngeal anatomy and/or pathology are encountered. A number of agents have been used for sedation during AFOI, including benzodiazepines, ketamine, propofol, sevoflurane, remifentanil and dexmedetomidine (Dex). Different protocols for sedation have been shown to improve the success rate (1–5).

There are a number of advantages of using remifentanil (Rem) for AFOI. Firstly, Rem is an ultra-short acting drug with a constant half life. The drug exerts antitussive effects that help to prevent coughing with tracheal manipulation. In addition, Rem attenuates cardiovascular responses to airway manipulation (6).

Dex is a selective α_2_-adrenergic receptor agonist that exert anxiolysis and analgesia effects, without respiratory depression. The drug has been used for intraoperative sedation during surgery under regional anesthesia, for sedation to mechanically ventilated patients in intensive care units, and for sedation during procedures, including AFOI (7,8). A previous study demonstrated that Dex and Rem were effective for sedation in patients undergoing AFOI (9).

Good topical anesthesia is also key to successful AFOI. Thyrocricocentesis is a useful technique; however, in cases with huge tumors or wound infections in the neck area, thyrocricocentesis is contraindicated. Thus, a modified method, which involves using an epidural catheter threaded through the suction channel of a fiberoptic bronchoscope to allow the patient to be sprayed with lidocaine via an epidural catheter onto the glottis and below the vocal cords, can be a useful alternative. Furthermore, the modified AFOI technique produces less stimulation compared with the application of a coarse bronchoscope below the vocal cords. Previous studies used a fiberoptic bronchoscope to spray lidocaine onto the glottis and below the vocal cords, which was termed the spray-as-you-go technique (9,10,11). Another study also used a fiberoptic bronchoscope to spray onto the glottis and thyrocricocentesis below the vocal cords (12). In the present study, a modified AFOI technique involving only a 1.1 mm three-orifice epidural catheter to spray lidocaine onto the glottis and below the vocal cords was used. This avoided cricothyroid membrane injection and the use of a coarse bronchoscope below the vocal cords. Thus, the present study investigated the efficacy of a modified AFOI method in cases with anticipated difficult airways. In addition, the efficacy of Rem and Dex as adjuvants were compared.

## Materials and methods

### Ethical approval

Following approval from the Institutional Review Board of the First Affiliated Hospital of Guangzhou University of Chinese Medicine (Guangzhou, China), written informed consent was obtained from each patient during the year 2013. The study was also registered as a clinical trial (http://www.clinicaltrials.gov, identifier: ChiCTR-TRC-13003151).

### Patients

A total of 90 adult patients with an American Society of Anesthesiologists classification of grade I–II underwent a modified AFOI procedure following airway evaluation. Patients were excluded from the study if they were pregnant, under the age of 18 years, had undergone emergency surgery, had an allergy to any of the drugs used or were unable to communicate effectively. In addition, patients were excluded if the surgeon requested nasal intubation, if the patient refused and/or if the patients were receiving long-term opioids or sedative medication.

### Intubation procedure

An experienced consultant anesthetist, who was certified in advanced airway life support, performed the airway management for all the study subjects. While one resident performed fiberoptic intubation, an additional resident controlled the drug infusion. Anesthetic data and postoperative follow-ups were documented by a study nurse. Intubation conditions were graded by the consultant anesthetist who performed the fiberoptic intubation. The intubating anesthetist, patients and the study nurse who recorded the details of the procedures were all blinded to the study.

The patients were randomized by pharmacy into one of two groups, which included the Rem and Dex groups. The patients were randomized at a ratio of 1:1 using a covariate adaptive randomization algorithm. Study drugs [1 mg remifentanil, intravenous (IV), Yichang Humanwell Pharmaceutical Co., Ltd., Yichang, China; and 200 mcg dexmedetomidine, IV, Jiangsu Hengrui Medicine Co., Ltd., Lianyungang, China] were prepared in accordance with the patient weight (kg), and were blinded to the anesthesia care team (consultant and resident) and the patients. All the residents had previously performed fiberoptic intubation at least 40 times.

Patients were informed and their consent was obtained by one of investigators at the preoperative evaluation one day prior to surgery. During the preoperative evaluation, an extensive airway examination was performed and the difficulty of the laryngoscopy or intubation procedure was assessed and assigned a simplified airway risk index (SARI) score of ≥4. The SARI score, as described by el-Ganzouri, comprised information regarding previous airway difficulties, the Mallampati classification, mobility of the neck, mouth opening, prognathism ability, the thyromental distance and the body mass index (kg/m^2^) (13).

The preparation of patients in each group was standardized as much as possible. Following pretreatment with intramuscular injections of 0.1 mg phenobarbital sodium and 0.5 mg atropine, each patient was moved to the operating room where an electrocardiogram, a non-invasive blood pressure cuff, a respiratory rate and a pulse oximeter were placed with a Philips monitor (Philips 865231; Philips Medizin Systeme Böblingen GmbH, Böblingen, Germany). The conscious level of the patient was evaluated using ‘state entropy monitoring’ (Datex-Ohmeda, Helsinki, Finland).

### Anesthesia application

Study drugs were administered in a 50-ml syringe as 200 μg Dex (2 ml) in 48 ml saline (0.9%), or in a 20-ml syringe as 1 mg Rem in 20 ml saline (0.9%). Patients in the Rem group received a loading dose of 0.75 μg/kg infused at 0.15 μg/kg/min over 5 min, followed by a continuous infusion of 0.1 μg/kg/min. Patients in the Dex group received a loading dose of 1 μg/kg infused over 10 min, followed by a continuous infusion of 0.3 μg/kg/h.

**Figure 1:**
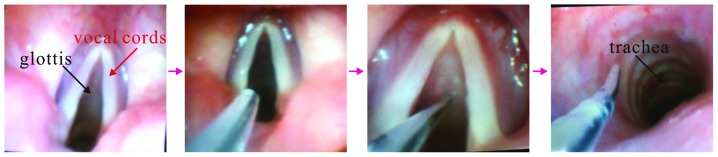
Images show the anesthesiologist spraying lidocaine (2%) via an epidural catheter onto the patient’s glottis and below the vocal cords.

Simultaneously, topical anesthesia was applied. Patients were asked to keep the lidocaine in their mouth for as long as possible before swallowing. The patients were administered 4 ml lidocaine (2%) via a laryngeal anesthesia catheter through the oral cavity and pharynx to reduce the gag reflex. While waiting for the desired level of sedation to be achieved, an epidural catheter was threaded through the suction channel of a fiberoptic bronchoscope (FOB; PENTAX FB-15RBS; Pentax Medical, Tokyo, Japan), which had an outer diameter of 4.8 mm. A longer (3–4 cm) flexible fiberoptic was applied in order for the patients to be sprayed with 4 ml lidocaine (2%) via an epidural catheter onto the glottis and below the vocal cords. This procedure was referred to as modified topical anesthesia, and avoided cricothyroid membrane injection and the application of a coarse bronchoscope below the vocal cords (Fig. 1).

### Anesthesia assessment

During the procedure, the anesthesiologists used the Ramsay Sedation Scale (RSS) to assess the level of sedation of the patients. If the RSS was <2, rescue doses of up to 20 mg propofol were administered. In the two groups, drug infusion was discontinued following successful intubation and general anesthesia was induced with 1–2 mg/kg IV propofol (Precedex, 200 mg/20 ml; Corden Pharma S.p.A. Caponago, Italy) and maintained with 4–5 mcg/kg IV fentanyl (Precedex, 100 mcg/2 ml; Yichang Humanwell Pharmaceutical Co., Ltd.), 1–2% end-tidal isoflurane (Maruishi Pharmaceutical Co., Ltd., Osaka, Japan) and 1 mg/kg IV vecuronium (powder; 4 mg; Hainan Star Pharmaceutical Co., Ltd., Haikou, China) for muscle relaxation. Fiberoptic intubation was initiated once the RSS reached a score of two. The outer diameter (OD) 4.8-mm FOB was loaded with an inner diameter (ID) 7.5-mm Parker Flex-Tip tube (#215075H; Well Lead Medical Instrument Co., Ltd., Guangzhou, China) for male patients, or an ID 7.0-mm Parker Flex-Tip (#215070H; Well Lead Medical Instrument Co., Ltd.) for females. An assistant performed a jaw thrust to expand the oropharyngeal space. Endotracheal tube placement was confirmed with capnography and bilateral auscultation. The primary endpoint was the time to tracheal intubation (TTI), as confirmed by capnography and measured by the advancement of the flexible fiberscope behind the teeth until the appearance of a capnography curve.

### Clinical outcome assessment

Primary outcomes measured included the intubation scores, as assessed by coughing (1, none; 2, slight; 3, moderate; and 4, severe) and limb movement (1, none; 2, slight, 3, moderate; and 4, severe). Secondly, patient tolerance was assessed by intubation comfort scores (1, no reaction, no change or a single change in the facial expression; 2, slight reaction, grimacing facial expressions; 3, moderate reaction, severe facial grimace but retained ability to follow verbal command and no reflex head movements; 4 severe reaction, severe facial grimace associated with head movements, but patient remains able to obey verbal commands; 5, very severe reaction, severe facial grimace associated with protective head and limb movements hindering the procedure and an inability to obey any verbal command; 6, uncooperative) (14). Furthermore, a three-point scale was used to assess the clinical outcome immediately following the tracheal intubation (1, cooperative; 2, restless with minimal resistance; 3, severe resistance with immediate application of general anesthesia). The lower the score, the better the patient condition. Once tracheal intubation was complete and the tracheal tube was secured, general anesthesia was administered.

Additional anesthetic parameters associated with the modified AFOI method included the conscious level (state entropy value and RSS level), airway obstruction score (1, patent airway; 2, airway obstruction relieved by neck extension; 3, airway obstruction requiring jaw retraction) and the consumption of the study drugs. In addition, the intubation time (time period between FOB insertion and the confirmation of tracheal intubation), the hypoxic episode (SpO_2_ of <90%) and the use of rescue doses for conscious level support were recorded. Hemodynamic changes (heart rate and mean arterial blood pressure) were compared between the two groups at five time points during the modified AFOI procedure, including at the baseline (preanesthetic preparation), at infusion (immediately prior to fiberoptic intubation), at intubation and at 1 and 5 min after tracheal intubation.

A postoperative follow-up was conducted the day following surgery, and amnesia (memory of preanesthetic preparations, topical anesthesia, endoscopy and intubation), incidence of adverse events (hoarseness and sore throat) and satisfaction score (1, excellent, 2, good, 3, fair, 4, poor) were assessed.

### Statistical analysis

SPSS 16.0 software (SPSS, Inc., Chicago, IL, USA) was used for statistical analysis. Normally distributed and continuous variables are presented as the mean ± standard deviation or mean ± standard error of the mean. Continuous data were compared using an unpaired t-test, while the Mann-Whitney U test was used to compare non-continuous data and non-normally distributed data. Intragroup comparisons of hemodynamic data at the various time points were performed using repeated measures analysis of variance. Where statistical significance was determined, Fisher’s protected least significant difference post hoc test was applied. The χ^2^ test or Fisher’s exact test were used to compare the categorical data between the two groups. Sample size calculation was based on a pilot study. P<0.05 was considered to indicate a statistically significant difference.

## Results

### Patient clinical data

In total, 90 patients completed the study. No statistically significant differences were observed between the baseline data of the two groups (Table I). The modified SARI score was 4.5±2.7 in the Rem group and 4.3±2.6 in the Dex group (Table I). SARI scores represented the sum of the individual risk factor weightings. All of the patients had no history of anesthesia usage; however, the preoperative interview indicated a suspicion of a difficult laryngoscopy intubation.

### Assessment of intubating conditions

All the patients underwent a successful fiberoptic intubation. As shown in Table II, three patients in the Rem group and two patients in the Dex group required a rescue infusion of propofol to achieve adequate sedation. The mean time to achieve sedation with Rem was 531.2 sec, while for Dex, the mean time was 673.1 sec. Thus, the TTI was lower in the Rem group compared with the Dex group. During endoscopy insertion, the Rem group exhibited more favorable intubation scores with regard to coughing. However, no statistically significant differences were observed in the sedation scale, intubation times and patient reactions when comparing the two groups.

### Adverse events in patients during procedure

In total, four patients from the Rem group and three patients in the Dex group were associated with severe airway obstruction, with five patients from the Rem group and four individuals from the Dex group developing transient hypoxia (Table III). An additional patient from each group exhibited transient hypoxia due to a sensitivity to the drugs. In the Rem group, the SpO_2_ values of the patients decreased to 88–90%, while their respiratory rates decreased to 8 or 9 bpm. Four patients in the Dex group had SpO_2_ values that decreased to 88%, with the respiratory rate decreasing to 10 bpm. Loud auditory stimuli and high-flow mask oxygen (8 l/min) were required to resolve the transient hypoxia. The mean respiratory rate with Rem was 12 bpm, and 11 bpm with Dex. No serious complications occurred in the two groups throughout the AFOI procedures.

### Hemodynamic level

**Figure 2:**
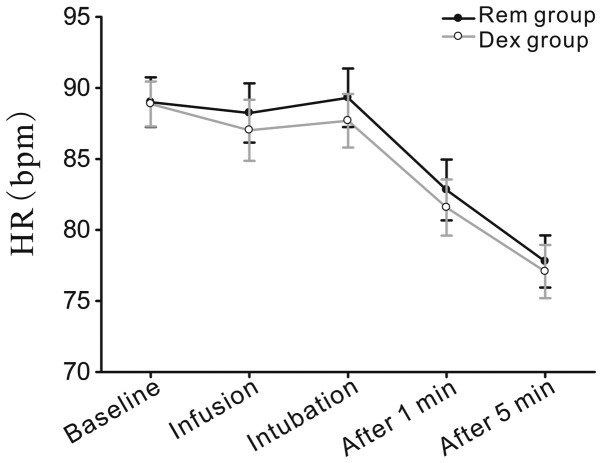
Changes in the HR of the patients receiving Dex or Rem during intubation. Hemodynamic parameters were analyzed at the baseline (preanesthetic preparation), at infusion (immediately prior to fiberoptic intubation), at intubation and at 1 and 5 min after tracheal intubation. No statistically significant differences were observed in the HR at the five points between the two groups. Data are expressed as the mean ± standard error of the mean. HR, heart rate; Dex, dexmedetomidine; Rem, remifentanil.

**Figure 3:**
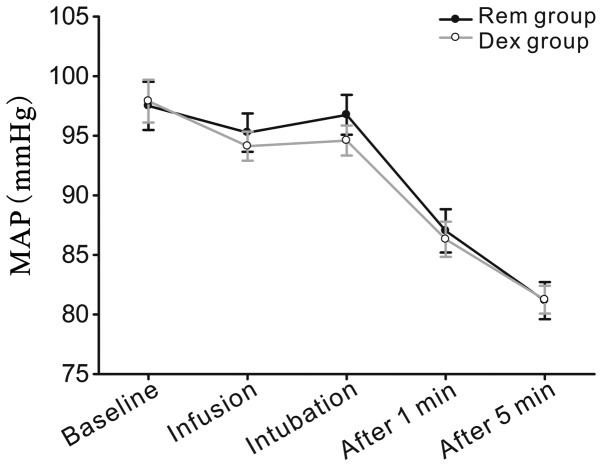
Changes in the MAP of the patients receiving Dex or Rem during intubation. Hemodynamic parameters were analyzed at the baseline (preanesthetic preparation), at infusion (immediately prior to fiberoptic intubation), at intubation and at 1 and 5 min after tracheal intubation. No statistically significant differences were observed in the MAP at the five points between the two groups. Data are expressed as the mean ± standard error of the mean. MAP, mean arterial blood pressure; Dex, dexmedetomidine; Rem, remifentanil.

Heart rate and mean arterial pressure were evaluated at five time points, as shown in Figs. 2 and 3. No statistically significant differences were observed between the predicted mean values for the Rem and Dex groups.

### Postoperative follow-up

A postoperative follow-up examination was conducted the day following surgery, and the interview parameters are shown in Table IV. With regard to patient tolerance, the lowest median (interquartile range) comfort score during the procedure was 2 (1–2) for the Dex and Rem groups (P>0.05). The levels of memory for preanesthetic events, topical anesthesia, endoscopy and intubation were 82.2, 57.8 and 26.7%, respectively, in the Dex group compared with 88.9, 62.2 and 31.1%, respectively, in the Rem group (P>0.05). The occurrence of postoperative adverse events did not differ significantly between the two groups (Table III).

## Discussion

The primary aims of the present study were to determine whether there were differences in the safety and efficacy of using remifentanil (Rem) and dexmedetomidine (Dex) regimens for sedation during modified AFOI. The modified AFOI method uses an improved topical anesthesia technique, where the drug is sprayed into the airway through an epidural catheter that has been passed through the suction channel of a FOB (15). The modified procedure avoids cricothyroid membrane injections and coarse bronchoscope application below the vocal cords (Fig. 1), which is particularly important for patients with large neck tumors, infection or who are unable to bear the stimulation of the coarse bronchoscope.

The current study demonstrated relatively similar efficacy of Rem and Dex as adjuvants to modified AFOI. During endoscopy insertion, the Rem group exhibited more favorable intubation scores with regard to coughing. However, there were no statistically significant differences in the sedation scale, intubation times and the patient reaction in the two groups. Recently, Hu *et al* (9) compared Rem and Dex treatment and demonstrated that Dex therapy results in improved endoscopy scores. The two differing results may due to the topical anesthesia applied. Hu *et al* advanced the tip of the FOB into the site below the glottis to prevent the FOB from slipping out of the trachea when spraying the endotracheal region, as a result of coughing or movement. However, in the present study, an epidural catheter was used for translaryngeal spraying of lidocaine, since a previous study had demonstrated that this method can produce a more effective airway topical anesthesia for fiberoscopy due to the more proximal site in the airway (12). The technical spreading of lidocaine through cricothyroid membrane injections is achieved primarily by the coughing of the patient immediately after the injection. Xue *et al* hypothesized that it was impossible to ensure that lidocaine was well-distributed along the infraglottic area and tracheal wall using this method (11).

During AFOI, it is crucial that the patient is relaxed and cooperative. Thus, conscious sedation is important. A number of studies have reported success with various agents, including Rem, Dex, midazola, propofol and ketamine. However Rem and Dex have been demonstrated to be more efficient compared with others (1–9).

Rem provides profound analgesia, suppresses airway reflexes and has minimal effect on cognitive function (6). These characteristics make the drug useful as an adjunct and also as a primary agent to provide sedation during AFOI (2,5,7,9,13,12). Vennila *et al* (16) revealed the mean effective site concentration for Rem as 6.3±3.87 ng/ml during nasal endoscopy and 8.06±3.52 ng/ml during tracheal intubation, as a single agent without the use of other sedatives/premedication and/or spray-as-you-go local anesthesia.

Dex activates the postsynaptic α_2_-adrenergic receptors in the locus coeruleus, and induced conscious sedation involves activation of the endogenous sleep-promoting pathway. In addition, Dex exhibits analgesic, anxiolytic and antisialagogue properties (17,18). Chu *et al* (10) demonstrated that combining Dex loading with topical anesthesia provides a significant benefit for AFOI with regard to intubation conditions, patient tolerance and hemodynamic parameters.

In the present study, the patients in the Dex group had a delayed intubation start time, possibly due to the different mechanisms of sedation between the two agents. The optimum sedation dose of Dex for AFOI has not been established, although a loading dose between 0.4 μg/kg and 1 mg/kg over a minimum of 10 min has been used to attain sedation. Cattano *et al* selected lower loading doses, which resulted in insufficient sedation and analgesia for a successful first attempt at AFOI (19). In the present study, a relatively high loading dose of 1 μg/kg over 10 min was applied, followed by a lower infusion rate of 0.3 μg/kg/h. The optimum drug dose for a sedative to achieve a careful balance of airway relaxation versus collapse is difficult to ascertain. Patients in the Rem group received a loading dose of 0.75 μg/kg infused at 0.15 μg/kg/min over 5 min, followed by a continuous infusion of 0.1 μg/kg/min. An RSS score of two was achieved almost immediately following the loading dose of the two groups. Dex administration achieved an RSS score of two at a slower rate compared with Rem. Four patients in the Rem and three patients in the Dex groups experienced severe airway obstruction, with five patients from the Rem and four patients from the Dex groups developing transient hypoxia (Table III). The further patient from each group exhibited transient hypoxia due to a sensitivity to the drugs. An additional patient in the Rem group exhibited transient hypoxia due to sensitivity to the drug. Loud auditory stimuli and high-flow mask oxygen (8 l/min) were required to resolve transient hypoxia. In the study by Scher and Gitlin, 1 μg/kg Dex was applied; however, a 15-mg ketamine bolus was also administered, followed by an infusion at 20 mg/h to achieve excellent intubating conditions for AFOI, including satisfactory sedation, patient cooperation and a dry airway (20). By contrast, Belda *et al* stated that addition of ketamine to Rem target-controlled infusion did not offer any advantages, and ketamine administration alone was not adequate sedation for AFOI (5).

With regard to hemodynamic stability, no statistically significant differences were observed in the mean arterial pressure and heart rate during intubation for the Dex or Rem groups. Hu *et al* reported statistically significant differences in the heart rate at the end of endoscopy and intubation, but no significant differences in the mean arterial pressure between the Rem and Dex groups at any time point (9). However, Hu *et al* used larger doses of Dex compared with the present study. The difference in results between the study by Hu *et al* and the present study may be due to the method of local anesthesia used as the modified AFOI technique in the current study used only a 1.1 mm three-orifice epidural catheter onto the glottis and below the vocal cords. It avoided using cricothyroid membrane injection and a coarse bronchoscope below the vocal cords, which may have reduced the release of noradrenaline (21). Dex infusion may cause adverse effects, including hypotension, hypertension, nausea, bradycardia, atrial fibrillation and hypoxia (22). In the present study, a SpO_2_ of <90% was observed in two patients from the Dex group and three patients in the Rem group. The symptom was easily managed by asking the patients to force inspiration, and the condition of all the patients improved. The intubation time and postoperative sore throat and hoarseness did not differ significantly between the two groups.

In the results of the present study, state entropy values of the two groups revealed consciousness levels comparable with clinical observation at tracheal intubation (state entropy, ~89; Table II). Patients receiving the Rem infusion revealed a comparative incidence of amnesia to topical anesthesia (88.9 vs. 86.7%), endoscopy (62.2 vs. 57.8%) and intubation (31.1 vs. 26.7%) when compared with the Dex group. In the present study, Dex loading did not decrease the state entropy values to a greater extent compared with Rem.

In conclusion, the Rem and Dex regimes utilized in the present study provided satisfactory intubating conditions and patient satisfaction in the majority of patients undergoing the modified AFOI procedure. Comparable upper airway patency to awake patients was observed, and only temporary hemodynamic adverse effects occurred in the patients. These properties indicate that Dex and Rem are useful drugs for providing conscious sedation; however, one limitation is that their administration may be associated with a greater incidence of recall.

The present study demonstrated that modified AFOI is a feasible and effective method for dealing with difficult airways, while Rem and Dex administration for sedation exert a similar efficacy.

## Figures and Tables

**Table I:** Demographic data of the patients.

Characteristics	Rem group	Dex group
Age, years	45.9±11.7	41.5±12.8
Weight, kg	61.9±11.1	63.6±11.0
Height, cm	162.72±6.5	166.5±7.1
BMI, kg/m^2^	23.5±3.3	22.9±3.4
ASA status	1.7±0.5	1.6±0.5
Modified SARI	4.5±2.7	4.3±2.6

Values are expressed as the mean ± standard deviation (n=45). ASA, American Society of Anesthesiologists; SARI, simplified airway risk index; Dex, dexmedetomidine; Rem, remifentanil; BMI, body mass index.

**Table II:** Anesthetic data during the modified AFOI procedure.

Intubation scores	Rem group	Dex group
Cough, 1/2/3/4, n	23/17/4/1^a^	19/16/7/3
Movement, 1/2/3/4, n	23/13/7/2	21/14/7/3
Intubation time, sec	52.0±20.2	50.1±28.3
Drug requirements, μg	137.4±47.6^a^	61.4±15.2
RSS at intubation	2.2±0.7	2.3±0.6
State entropy at intubation	88.1±0.7	89.2±1.1
Rescue requirement for consciousness, n (%)	3 (10.0)	2 (6.7)
Time to tracheal intubation, sec	531.2±7.2^a^	673.1±8.3

Data are expressed as the mean ± standard deviation, or as a number and percentage (n=45).

a P<0.05, vs. Rem group;

AFOI, awake fiberoptic orotracheal intubation; RSS, Ramsay Sedation Scale; Dex, dexmedetomidine; Rem, remifentanil.

**Table III:** Adverse events in patients receiving Rem or Dex during modified AFOI.

Adverse event	Rem group	Dex group
Airway obstruction score, 1/2/3, n	34/7/4	35/7/3
Hypoxia, n (%)	5 (11.1)	4 (8.9)
Respiratory rate, bpm	12±3.4	11±3.9

Data are expressed as the mean ± standard deviation or as a number and percentage (n=45). Dex, dexmedetomidine; Rem, remifentanil; AFOI, awake fiberoptic orotracheal intubation.

**Table IV:** Postoperative follow-up data.

Follow-up parameters	Rem group	Dex group
Sore throat, n (%)	10 (22.2)	11 (24.4)
Hoarseness, n (%)	3 (6.7)	2 (4.4)
Satisfaction score (1–4)	2 (1–2)	2 (1–2)
Recall of topical anesthesia, n (%)	40 (88.9)	37 (82.2)
Recall of endoscopy, n (%)	28 (62.2)	26 (57.8)
Recall of intubation, n (%)	14 (31.1)	12 (26.7)

Data are expressed as the median (interquartile range) or as a number and percentage (n=45). Dex, dexmedetomidine; Rem, remifentanil.

## Abbreviations

AFOI: awake fiberoptic tracheal intubation
Rem: remifentanil
Dex: dexmedetomidine
TTI: time to tracheal intubation
SARI: simple airway risk index
FOB: fiberoptic bronchoscope
RSS: Ramsay Sedation Scale
